# The Impact of Nurse-Led Cardiac Rehabilitation on Quality of Life and Biophysiological Parameters in Patients With Heart Failure: A Randomized Clinical Trial

**DOI:** 10.1097/JNR.0000000000000407

**Published:** 2020-10-08

**Authors:** Porkodi ARJUNAN, Ramakrishnan Venkatakrishnan TRICHUR

**Affiliations:** 1 PhD, RN, Reader, Faculty of Nursing, Sri Ramachandra Institute of Higher Education & Research (DU), Chennai, India; 2 MD, Professor, Department of Emergency Medicine, Sri Ramachandra Institute of Higher Education & Research (DU), Chennai, India.

**Keywords:** biophysiological parameters, heart failure, nurse-led cardiac rehabilitation, quality of life, patients with chronic disease

## Abstract

**Background:**

Cardiovascular diseases are the leading cause of mortality in the Indian subcontinent, accounting for 38% of deaths annually. One cardiovascular disease in particular, heart failure, is a growing public health problem both in India and worldwide.

**Purpose:**

Heart failure is a chronic, progressive disease with increasing rates of incidence and prevalence. This study was conducted to determine the influence of a nurse-led cardiac rehabilitation program on quality of life and biophysiological parameters in patients with chronic heart failure. In this study, it was hypothesized that participants in the cardiac rehabilitation program would report significantly more-positive changes in quality of life and biophysiological parameters than their peers who did not participate in this program.

**Methods:**

In this randomized controlled trial, the participants were patients with chronic heart failure who had been admitted to a tertiary care hospital in India. The participants assigned to the intervention group received both nurse-led cardiac rehabilitation and routine care. In addition, intervention group participants received a booklet on cardiac rehabilitation, *Healthy Way to Healthy Heart*, at discharge and fortnightly telephone reminders about good cardiac rehabilitation practices. A standard questionnaire was used to collect targeted information on participants' general and disease-specific quality of life at 1 and 3 months postintervention. Biophysiological parameters such as body mass index, blood pressure, and serum cholesterol values were also measured.

**Results:**

Two thirds of the participants in each group (65% in the intervention group and 66% in the control group) were between 51 and 70 years old. The mean score for the mental component summary of generic quality of life steadily decreased in the control group and steadily increased in the intervention group at the first and second posttests.

**Conclusions/Implications for Practice:**

Nurses working in cardiology units play a pivotal role in educating and managing the health status of patients with heart failure. Providing cardiac rehabilitation to patients with heart failure benefits the quality of life of these patients. Nurses working in cardiology units should encourage patients with heart failure to practice cardiac rehabilitation for a longer period to further improve their quality of life.

## Introduction

Cardiovascular diseases (CVDs) are the leading cause of death worldwide, and in Southeast Asia, 38% of all deaths occur before the age of 70 years (World Health Organization, 2015). Heart failure (HF) is a CVD that is a growing public health problem both in India and worldwide (Nagai et al., 2016). Globally, HF affects approximately 26 million people (Ambrosy et al., 2014). In India, it has been reported that HF affects 1.3–4.6 million individuals, with an annual incidence of 0.5–1.8 million (Population Division, Department of Economic and Social Affairs, United Nations, 2013). In addition, over 5.1 million people live with HF in developed countries such as the United States of America (Hobbs et al., 2016), and this number is expected to increase to more than eight million by 2030 (Heidenreich et al., 2011).

HF is a syndrome of many CVDs, including coronary artery disease, hypertensive heart disease, dilated cardiomyopathy, and valvular heart disease (Felker et al., 2003). Therefore, the rising incidence of cardiac diseases that cause HF is increasing the rates of chronic HF (CHF) mortality and morbidity (Heart Failure Society of America, 2010). The effective management of HF depends on the related underlying cause. Hence, there are multiple combinations of treatments for HF, including medical, surgical, and supportive management (Shaffer & Maurer, 2015).

Quality of life (QoL) is an important outcome indicator for patients with CVD (Amedro et al., 2016; Teixeira et al., 2011). Most patients with HF experience changes in functional abilities that negatively affect QoL (Hong, 2015). Thus, it is important that these patients adapt to the chronic nature of their condition by having thorough knowledge about this disease and its management. The common factors that affect QoL in patients with HF include attitudes toward following cardiac rehabilitation (CR), knowledge about the disease, and the current stage of the disease (Evangelista et al., 2010; Nesbitt et al., 2014).

The effective management of CHF also requires controlling the risk factors and lifestyle habits that are known to worsen the disease. For instance, decreased level of high-density lipoproteins (HDL) is a risk factor for cardiovascular events (Forti & Diament, 2006). A significant association between increased cholesterol level and poor outcomes in patients with CHF has been highlighted in prior studies (Kalantar-Zadeh et al., 2004). Furthermore, poor prognosis has been reported in patients with CHF because of decreased serum sodium level (Rusinaru et al., 2012), decreased hemoglobin level in the blood, and low ejection fraction (Ezekowitz et al., 2003; Tang & Katz, 2006). In addition, other physiological parameters such as decreased body mass index (BMI) have been associated with increased mortality in patients with CHF (Pocock et al., 2005).

CR is a major component of the critical management required by patients with CHF. Therefore, it is important to teach patients with CHF regarding the nature of this disease and the associated behavioral and lifestyle modifications necessary to achieve effective management. Educating patients with CHF is most effective when it is tailored to patient age group and disease status (Schopfer & Forman, 2016). CR has been reported in many studies to have a positive effect on QoL in patients with HF (Conway, 2015). On the basis of the literature, topics such as etiology, pathophysiology, signs and symptoms, pharmacological treatment, lifestyle modification, diet and exercise, sexual activity, sleep and breathing disorders, adherence, psychosocial aspects, and disease prognosis (McMurray et al., 2012) should be included in educational programs designed for patients with HF.

The objective of this study was to evaluate the effect of a nurse-led CR program on QoL and biophysiological parameters in patients with HF.

## Methods

### Study Design and Participants

This randomized controlled trial study was conducted at one tertiary care hospital in India. Two hundred patients hospitalized with HF and between 31 and 88 years old were enrolled and randomly assigned into either the intervention or control group using computer-generated block randomization. The English alphabet letters “A” (intervention group) and “B” (control group) were affixed to 10 blocks each. The resultant 20 blocks were sealed individually in sequentially numbered, opaque envelopes and locked in the principal investigator's office cupboard. The sample size for this study was calculated based on a previous study (Austin et al., 2005). Considering QoL as the primary outcome, the estimated sample size was 180, which achieved a power of 80% at a level of significance at .05. A 10% attrition rate was estimated. Thus, a sample size of 200 was recruited, with 100 in each group. The study participants were blinded to group assignment. Inclusion criteria included > 31 years old, a medical diagnosis of HF with ejection fraction less than 40%, and dyspnea Grades II and III based on the New York Heart Association classification. Exclusion criteria included having undergone surgery as a part of the treatment for HF; experiencing complications such as arrhythmias, peripheral vascular disease, cerebrovascular accident, and end-stage renal disease; having a history of congenital heart disease; and having a treatment plan that involves ventricular assistive devices.

### Measurement

The demographic variables of the participants were assessed using a structured questionnaire. General QoL and disease-specific QoL were assessed using the following standard questionnaires: Medical Outcomes Study Questionnaire Short Form 36 Health Survey (SF-36) and the Minnesota Living with Heart Failure Questionnaire (MLHFQ). The SF-36 was used to assess functional health and well-being scores as well as physical and mental health summary measures. This tool comprises 36 items that are each scored using a 5-point Likert scale. The physical component summary of the SF-36 includes the four domains of physical functioning, role functioning physical, bodily pain, and general health perceptions, whereas the mental component summary includes the four domains of vitality, social functioning, role functioning emotional, and mental health. A higher total score on the SF-36 indicates better QoL. The reliability of the SF-36 was previously measured as .90.

Disease-specific QoL was measured using the MLHFQ. This tool includes 21 items covering HF-related physical, psychological, and social impairments in three domains, including physical (eight items), emotional (five items), and global (all 21 items). Each item is rated on a scale of 0–5, with higher scores indicating poorer QoL. The reliability of the MLHFQ was previously measured as .87. The biophysiological measures considered in this study included BMI, systolic and diastolic blood pressure (BP), lipid profile, hemoglobin, random blood sugar, serum sodium, and potassium.

### Intervention

After conducting the pretest, the nurse-led CR program, consisting of structured teaching about the disease condition, diet, exercise, medication, home care instruction, smoking cessation, and lifestyle modification, was administered on a one-to-one basis in three different sessions at the bedside of the intervention group. The intervention group was further required to participate in a 6-minute walk test. A booklet, *Healthy Way to Healthy Heart*, was provided to the intervention group on the day of discharge. In addition, telephone calls were made to each intervention group participant once every 2 weeks over the 3-month posttest period. Posttest 1 data were collected 1 month after the intervention, and Posttest 2 data were collected 3 months after the intervention. The participants in the control group received routine care only (e.g., physician's visits, nursing care, physiotherapy) and received the booklet *Healthy Way to Healthy Life* after providing Posttest 2 data.

### Data Collection Procedures

**Figure 1.**
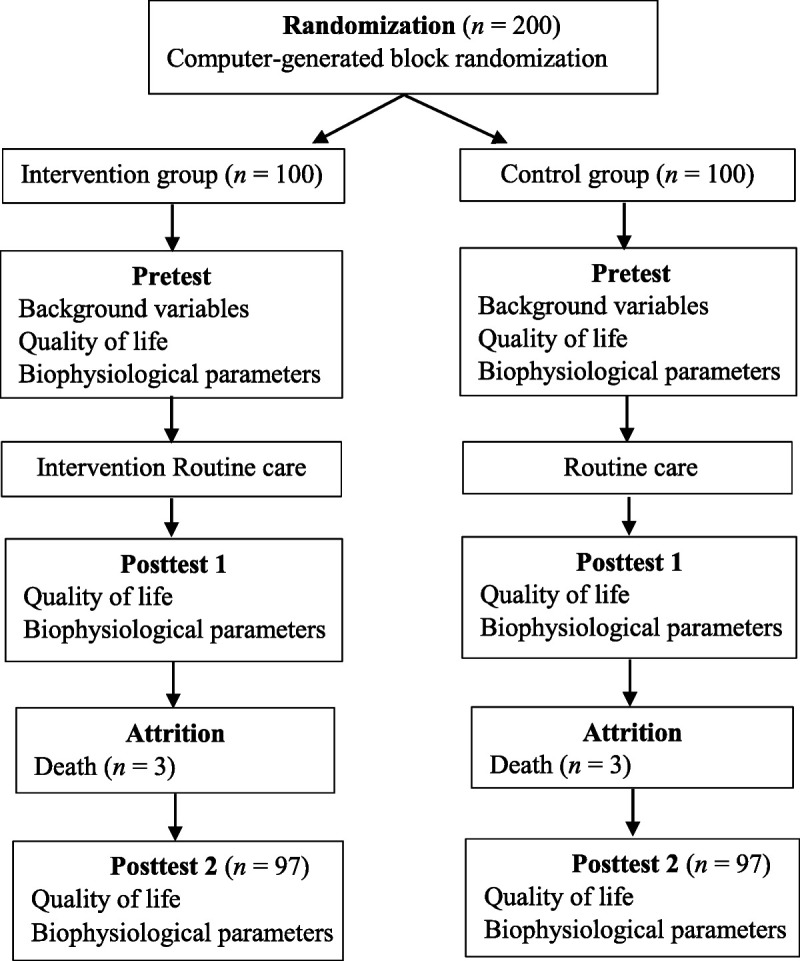
A Flowchart: Data Collection Procedure

The data were collected from January 8, 2011, to October 2, 2014. The institutional review board of Sri Ramachandra Medical College & Research Institute (Deemed University) approved this study (IEC[NI]/07/Nov/01/03). The principal investigator explained to the participants the risks and benefits of the study and the confidentiality of the data as well as answered questions before obtaining informed consent. The demographic variables of the study participants were collected via one-to-one interviews and medical records. QoL information was collected from the participants using the two questionnaires (SF-36 V2 and MLHFQ), and biophysiological parameters were collected from medical records. After confirming appointments with intervention group participants, the investigator visited them at their bedside and taught them CR for 3 consecutive days using a laptop. The booklet *Healthy Way to Healthy Life* was given to intervention group participants at discharge, and they were contacted by the investigator once every 2 weeks over the 3-month posttest period as part of the reinforcement process. The pretest data collection was conducted on the second day of hospital admission. Posttest 1 was conducted 30 days after completion of the intervention, with completed questionnaires collected by the investigator at each participant's 1-month follow-up clinical visit. Posttest 2 was conducted 90 days after completion of the intervention, with completed questionnaires collected by the investigator at each participant's 3-month follow-up clinical visit (Figure 1).

### Statistical Analysis

Continuous variables are presented in this study as mean and standard deviation, whereas categorical variables are presented as frequencies and percentages. A paired *t* test was conducted to identify the effect of the structured teaching program on QoL and biophysiological parameters. An independent *t* test was conducted to compare differences in variable values between the intervention and control groups. A nonparametric test such as the Wilcoxon signed ranks test and Mann–Whitney test was conducted when a nonnormal distribution of a mean value greater than 2 times of the standard deviation was observed. Repeated-measures analysis of variance was used to compare the intervention effect among pretest, Posttest 1, and Posttest 2. For all of the statistical tests, a *p* value of < .05 was considered significant. All of the statistical analyses were performed using SPSS Version 22.0 (IBM, Inc., Armonk, NY, USA).

## Results

### Demographics of the Study Participants

Two hundred patients with HF were included as participants in this study. As shown in Table 1, most of the participants were in the 51- to 70-year age range in both the intervention group (65%) and the control group (66%), and over three quarters of the participants were male in both the intervention group (78%) and the control group (79%). Furthermore, no significant differences were identified between the two groups in terms of demographic characteristics, with the exception of monthly total family income (*p* = .028).

**Table 1.** Participants' Demographic Characteristics (*N* = 200)

	Intervention (*n* = 100)	Control Group (*n* = 100)		
Demographic Variable	*n*%	*n*%	χ^2^	*p*
Age (years; mean = 59.57, *SD* = 11.16)			1.18	.550
31–50	2222	1717		
51–70	6565	6666		
> 70	1313	1717		
Gender			0.03	.863
Male	7878	7979		
Female	2222	2121		
Level of education			0.88	.820
Nonliterate	1414	1010		
Primary school	2323	2222		
High school	4545	4949		
College	1818	1919		
Occupation			0.04	.980
Skilled	4444	4545		
Professional	2121	2020		
Retired	3535	3535		
Marital status			4.80	.187
Married	9696	9696		
Unmarried	44	11		
Separated/widowed	00	33		
Religion			2.63	.269
Hindu	9191	9090		
Christian	44	88		
Muslim	55	22		
Residence			1.85	.397
Urban	5050	4141		
Rural	1919	2525		
Semiurban	3131	3434		
Monthly total family income (USD)			9.08	.028*
≤ 59.35	4141	2222		
59.36–89.02	2121	2323		
89.03–118.70	1313	2121		
> 118.70	2525	3434		
Type of family			0.33	.565
Extended family	5757	6161		
Nuclear	4343	3939		
Tobacco use			0.99	.321
Yes	2121	2727		
No	7979	7373		

### Intergroup Comparison of Biophysiological Parameters at Pretest

The biophysiological parameter data for the two groups at pretest are shown in Table 2. The mean scores for BMI were within the normal range for both groups, and the mean values for low-density lipoprotein values were at the optimal level for both groups. The HDL mean scores for the intervention group (33.91) and control group (35.12) indicate a high risk for heart disease. Random blood sugar level was above normal for both groups. Other biophysiological parameters, including systolic and diastolic BP, sodium, potassium, and hemoglobin, were within normal limits for both groups. Furthermore, no significant differences in biophysiological parameters were identified between the two groups at pretest.

**Table 2.** Biophysiological Parameters of the Intervention and Control Groups at Pretest (*N* = 200)

	Intervention Group (*n* = 100)		Control Group (*n* = 100)				
Biophysiological Parameter	Mean	*SD*	Mean	*SD*	Mean Difference	*t*	*p*
BMI (kg/m^2^)	24.29	3.02	24.94	3.92	0.65	1.31	.18
Systolic BP (mmHg)	124.22	22.10	125.12	16.47	0.90	0.32	.74
Diastolic BP (mmHg)	78.88	11.95	80.14	9.31	1.26	0.83	.40
LDL (mg/dL)	99.84	40.82	94.26	37.92	5.58	0.89	.37
HDL (mg/dL)	33.91	15.03	35.12	10.51	1.21	0.59	.55
Triglyceride (mg/dL)	121.08	73.53	132.72	72.62	11.64	1.35	.17
Hemoglobin (gm/dL)	12.28	2.33	12.27	2.07	0.01	0.03	.97
RBS (mg/dL)	174.20	97.09	176.70	107.97	2.49	0.40	.68
Serum sodium (mEq/L)	134.46	6.05	134.26	5.35	0.20	0.24	.80
Serum potassium (mEq/L)	4.17	0.77	4.10	0.73	0.07	0.72	.46

***Note.*** BMI = body mass index; BP = blood pressure; LDL = low-density lipoprotein; HDL = high-density lipoprotein; RBS = random blood sugar.

### Intergroup Comparison of Biophysiological Parameters at Posttest 2

The results summarized in Table 3 show significant differences in diastolic BP (*t* = 3.86, *p* < .001), HDL (*t* = 8.43, *p* < .001), hemoglobin (*t* = 2.31, *p* = .02), random blood sugar (*t* = 2.55, *p* = .01), sodium (*t* = 3.93, *p* < .001), and potassium (*t* = 5.01, *p* < .001) levels between the intervention and control groups after the intervention, with the intervention group showing significantly lower mean levels than the control group.

**Table 3.** Changes in Biophysiological Parameters Between the Intervention and Control Groups After the Intervention: Posttest 2 (*N* = 200)

	Intervention Group		Control Group				
Biophysiological Parameter	Mean	*SD*	Mean	*SD*	Mean Difference	*t*	*p*
BMI (kg/m^2^)	24.54	2.99	25.13	3.67	0.59	1.23	.22
Systolic BP (mmHg)	123.96	9.09	126.74	9.41	2.78	2.09	.38
Diastolic BP (mmHg)	80.39	5.77	83.63	5.88	3.23	3.86	< .001***
LDL (mg/dL)	90.24	6.07	87.62	23.36	2.61	1.06	.28
HDL (mg/dL)	49.46	6.18	41.00	7.71	8.46	8.43	< .001***
Triglyceride (mg/dL)	109.29	23.90	118.56	42.91	9.26	1.85	.06
Hemoglobin (gm/dL)	12.15	1.73	11.61	1.52	0.54	2.31	.02*
RBS (mg/dL)	137.71	34.74	153.67	5.67	15.95	2.55	.01**
Serum sodium (mEq/L)	139.90	3.90	137.91	3.09	1.99	3.93	< .001***
Serum potassium (mEq/L)	4.32	0.51	4.02	0.28	0.29	5.01	< .001***

***Note.*** BMI = body mass index; BP = blood pressure; LDL = low-density lipoprotein; HDL = high-density lipoprotein; RBS = random blood sugar.

*p < .05. **p < .01. ***p < .001.

### Impact on Quality of Life

The results presented in Table 4 show at Posttest 2 a significant improvement in the physical component (*t* = 2.23, *p* = .02), mental component (*t* = 11.17, *p* < .001), and disease-specific (*t* = 5.92, *p* < .001) QoL of the participants in the intervention group in comparison with the control group. Moreover, disease-specific QoL (*t* = 2.19, *p* = .02) improved more significantly in the intervention group than in the control group at Posttest 1.

**Table 4.** Changes From Baseline in Quality of Life Between the Intervention and Control Groups at Posttests 1 and 2 (*N* = 200)

	Intervention Group (*n* = 100)		Control Group (*n* = 100)				
Variable	Mean	*SD*	Mean	*SD*	Mean Difference	*t*	*p*
Physical component QoL							
Pretest	33.69	7.68	35.47	5.87	1.78	1.84	.06
Posttest 1	43.67	6.85	42.92	8.05	0.75	0.71	.47
Posttest 2	46.53	3.72	44.87	6.30	1.66	2.23	.02*
Mental component QoL							
Pretest	38.64	11.67	41.87	12.32	3.22	1.90	.05
Posttest 1	38.85	7.45	39.64	10.28	0.79	0.62	.53
Posttest 2	45.93	6.70	34.87	7.07	11.05	11.17	< .001***
Disease-specific QoL							
Pretest	48.51	24.30	43.70	20.99	4.81	1.49	.13
Posttest 1	35.37	14.47	40.12	16.07	4.75	2.19	.02*
Posttest 2	33.24	9.18	43.33	14.03	10.09	5.92	< .001***

***Note.*** QOL = quality of life.

*p < .05. ***p < .001.

### Result of Repeated Measure on Quality of Life

The results in Table 5 describe the changes from baseline in the physical, mental, and disease-specific QoL of the participants, indicating that significant interactions have a more favorable effect on the physical (*M* = 46.53, *SD* = 3.72), mental (*M* = 45.93, *SD* = 6.70), and disease-specific (*M* = 33.24, *SD* = 9.18) QoL in the intervention group than in the control group. The intervention group exhibited an increase in QoL during the second posttest.

**Table 5.** Repeated-Measures ANOVA on Quality of Life at Different Periods of Time Among the Intervention and Control Groups (*N* = 200)

	Pretest		Posttest 1		Posttest 2			
Quality of Life	Mean	*SD*	Mean	*SD*	Mean	*SD*	*F*	*p*
PCS							5.35	.020*
Intervention group	33.72	7.74	43.84	6.77	46.53	3.72		
Control group	35.61	5.78	43.27	7.88	44.87	6.31		
MCS							42.65	< .001***
Intervention group	38.60	11.59	38.93	7.02	45.93	6.70		
Control group	42.01	12.42	40.09	9.99	34.87	7.07		
Disease specific							12.26	.001***
Intervention group	48.09	24.46	34.86	13.62	33.24	9.18		
Control group	43.60	21.20	39.67	16.07	43.33	14.03		

***Note.*** ANOVA = analysis of variance; PCS = physical component summary; MCS = mental component summary.

*p < .05. ***p < .001.

## Discussion

The results of this study show that the CR educational program, which focused on improving disease knowledge, exercise, medication, and lifestyle habits, effectively improved QoL in the intervention group. The physical component of the generic QoL in the intervention group gradually increased from baseline through the second posttest. This suggests that following the rehabilitation program for a minimum of 3 months should improve physical QoL in patients with HF. The patients who received only routine care in the control group experienced a gradual decrease in the physical component of the generic QoL from baseline through the second posttest. These findings are consistent with Hasanpour-Dehkordi et al. (2016), who found that training, caring, and consultative nursing interventions improve QoL in patients with CHF.

In addition, the findings of this study indicate that this nurse-led CR program significantly and rapidly (within 1 month postintervention) improves the mental health components of QoL such as vitality, social functioning, role functioning emotional, and mental health. This suggests that there is a need to enhance physical care for problems common among patients with HF such as body pain and measures to improve self-care. It also further substantiates that receiving adequate knowledge regarding disease condition and lifestyle modification enhances the confidence of patients with HF to live a healthy life, thereby improving their functional abilities.

The baseline disease-specific QoL for both groups in this study was nearly equivalent, whereas the first and second posttest scores for disease-specific QoL improved significantly in the intervention group and decreased significantly in the control group. CR education, exercise, and improved 6-minute walk functional ability may contribute to the identified improvements in QoL in the intervention group. Cajanding (2016) reported similar findings in Filipino patients with HF who underwent cognitive behavioral therapy and reported significant improvements in QoL, self-esteem, and mood scores compared with the control (standard care) group.

### Biophysiological Parameters

Regarding the impact of the nurse-led CR on BMI, no significant difference was found between the two groups from pretest through the second posttest. The average BMI from pretest to the second posttest had increased in both the groups with a mean difference of 0.59, which, although not statistically significant, had clinical significance because the intervention group was within normal BMI whereas the BMI of the control group had increased. This may be attributable to dietary habit changes made after reading the booklet and to routine care by the dietician who visited both of the groups and discussed the diet plan.

The results showed no significant differences in biophysiological parameters such as BMI, BP, hemoglobin, random blood sugar, and serum electrolytes between the two groups during pretest. Moreover, diastolic BP level was within normal when compared with the control group. Therefore, the changes observed in diastolic BP occurred within the normal level. The 3-month follow-up period may not be sufficient to show significant changes in patients with HF, and it is possible that the mortality rate in the control group would increase. However, Tuomilehto et al. (1998) indicated that low diastolic BP is a significant predictor of cardiovascular mortality in the older adult (> 50 years old) population and that the risk of death from low diastolic BP and high systolic BP is higher in patients with CHF. These findings were not in agreement with this study.

Dyslipidemia significantly affected the control group, whereas posttest HDL levels were significantly improved in the intervention group. Potočnjak et al. (2016) found an association between low HDL cholesterol and increased risk for hospital mortality in acute patients with HF. BMI was calculated using the standard equation and expressed as kilograms per square meter (kg/m^2^). The results of this study indicate that CR education has minimal/no effect on BMI. However, Tevik et al. (2015) found that poor nutritional status almost doubled the risk of early mortality, independent of age and HF severity. The mean scores for BMI, systolic BP, low-density lipoproteins, and triglycerides for both groups indicate that biophysiological parameters fell within normal ranges. These findings are similar to Miller et al. (2011), who found that participants with a normal BMI (< 25 kg/m^2^) were more likely to have triglyceride levels < 150 mg/dl (43%) and < 200 mg/dl (39%).

### Implications for Practice

Nurses working in cardiology units should encourage patients with HF to practice CR for longer periods to improve their QoL. CR programs should be incorporated into the management plans for patients with HF to enhance QoL. Nurses may adopt telenursing strategies to overcome the physical problems that typically impeded postdischarge access to patients with HF. Long-term follow-up studies or cohort studies are necessary to assess the durability of the benefits of rehabilitation interventions in patients with HF.

### Limitations

This study was limited to a single center, and thus the generalizability of findings may be affected. Participation was limited to patients diagnosed with HF because of coronary artery disease, dilated cardiomyopathy, or hypertensive heart disease and with ejection fractions below 40%. Diagnoses of myocardial infarction and valvular heart disease leading to HF were excluded because the mode of treatment for these conditions is surgery.

### Conclusions

CHF affects the QoL of patients, with the most significant impacts affecting physical aspects such as physical functioning, role functioning physical, bodily pain, and general health perceptions and less significantly affecting mental health aspects such as vitality, social functioning, role functioning emotional, and mental health. Nurses involved in CR should focus on improving the physical functioning of patients with CHF.
